# MT1-MMP Expression Levels and Catalytic Functions Dictate LDL Receptor-Related Protein-1 Ligand Internalization Capacity in U87 Glioblastoma Cells

**DOI:** 10.3390/ijms232214214

**Published:** 2022-11-17

**Authors:** Jonathan Pratt, Khadidja Haidara, Borhane Annabi

**Affiliations:** Laboratoire d’Oncologie Moléculaire, Centre de Recherche CERMO-FC, Département de Chimie, Université du Québec à Montréal, Montréal, QC H3C 3P8, Canada

**Keywords:** blood–brain tumor barrier, MT1-MMP, LRP-1, glioblastoma, Angiopep-2

## Abstract

Modulations in cell surface receptor ectodomain proteolytic shedding impact on receptor function and cancer biomarker expression. As such, heavily pursued therapeutic avenues have exploited LDL receptor-related protein-1 (LRP-1)-mediated capacity in internalizing Angiopep-2 (An2), a brain-penetrating peptide that allows An2–drug conjugates to cross the blood–brain tumor barrier (BBTB). Given that LRP-1 is proteolytically shed from the cell surface through matrix metalloproteinase (MMP) activity, the balance between MMP expression/function and LRP-1-mediated An2 internalization is unknown. In this study, we found that membrane type-1 (MT1)-MMP expression increased from grade 1 to 4 brain tumors, while that of LRP-1 decreased inversely. MMP pharmacological inhibitors such as Ilomastat, Doxycycline and Actinonin increased in vitro An2 internalization by up to 2.5 fold within a human grade IV-derived U87 glioblastoma cell model. Transient siRNA-mediated MT1-MMP gene silencing resulted in increased basal An2 cell surface binding and intracellular uptake, while recombinant MT1-MMP overexpression reduced both cell surface LRP-1 expression as well as An2 internalization. The addition of Ilomastat to cells overexpressing recombinant MT1-MMP restored LRP-1 expression at the cell surface and An2 uptake to levels comparable to those observed in control cells. Collectively, our data suggest that MT1-MMP expression status dictates An2-mediated internalization processes in part by regulating cell surface LRP-1 functions. Such evidence prompts preclinical evaluations of combined MMP inhibitors/An2–drug conjugate administration to potentially increase the treatment of high-MT1-MMP-expressing brain tumors.

## 1. Introduction

Glioblastoma multiforme (GBM) is the most malignant and frequently occurring type of primary astrocytomas, and accounts for more than 60% of all brain tumors in adults [1]. It represents one of the most challenging cancers to treat due, in part, to the protection that the blood–brain tumor barrier (BBTB) provides and to its capacity to adapt to strict environmental conditions [2]. Thus, brain microvasculature plays a crucial role in sustaining GBM and, as a result, can be considered as a therapeutic target. Tumor angiogenesis leads to leaky vasculature compared to the normal blood–brain barrier (BBB) microvasculature that exhibits extremely low permeability to most blood-borne substrates. To further complicate the design of targeted therapeutic strategies, brain tumor molecular subtyping has revealed differential expression of myriad cell surface receptors and prognostic biomarkers within grade 1 to grade 4 that, combined, account for their increasing invasive phenotype [3,4]. Accordingly, recent drug delivery strategies targeting gliomas have used cell-mediated magnetic directing [5], receptor-mediated endocytosis of macromolecules [6], functional bionanoparticles [7,8], or targeted drug delivery into a specified cellular compartment [9]. Unfortunately, most have met with very limited success. Continued understanding of cancer cell surface biology and unraveling the roles of regulated macromolecular receptors in targeted drug delivery are therefore imperative to optimizing targeted therapies.

The targeting of tumor cells by antineoplastic drugs is, due to inter-tumoral molecular heterogeneity, often characterized by low selectivity. As a result, different types of natural and synthetic delivery systems have been proposed as carriers for improving the selectivity of antitumor drugs [10,11] and, in the case of brain cancer, for enhancing BBTB permeability to enable efficient GBM treatment [12,13]. While several receptors for hormones, growth factors, folic acid, and vitamin B12 are overexpressed in cancer cells [14], the overexpression of low-density lipoprotein receptors (LDLRs) in various tumor cells has been observed both in response to adaptive mechanisms and consequent to the large quantities of cholesterol and fatty acids required for supporting rapid proliferation [7]. This adaptive molecular signature was documented in seven glioblastoma cell lines that showed that these cells had high LDLR expression [15]. In addition, the expression and function of receptor low-density lipoprotein receptor-related protein-1 (LRP-1) was shown to correlate with glioblastoma cell invasion [15,16,17]. LRP-1 is a multifunctional endocytic recycling protein shuttling from the cell surface to intracellular compartments and back to the cell surface. LRP-1 ligand internalization functions were later exploited and found to very efficiently enable a 19 amino acid brain-penetrant peptide, named Angiopep-2 (An2), to cross the BBTB and to target the brain cancer cell compartment [18,19]. Conjugation of this peptide to small therapeutic molecules such as paclitaxel, doxorubicin or etoposide significantly increased their internalization across the BBTB upon systemic administration [20,21]. ANG1005, a brain-penetrant Angiopep–paclitaxel bioconjugate, has recently shown clinical activity for the treatment of leptomeningeal carcinomatosis and recurrent brain metastases in breast cancer patients [22].

In numerous physiological and pathological processes, including wound healing, embryogenesis, and tumor invasion, the extracellular matrix (ECM) is hydrolyzed by a family of enzymes known as matrix metalloproteinases (MMPs) [23]. The MT1-MMP, commonly known as MMP-14, was the first membrane-anchored MMP to be discovered and has received the most attention among the other MMPs. Although its extracellular catalytic domain has a role in numerous biological processes, including the remodeling of the ECM and the migration of cells engaged in angiogenesis, metastasis, and tissue invasion/infiltration [24,25], its recently documented intracellular signal transducing functions were found to signal apoptosis, autophagy, and inflammatory processes, all of which are associated with a therapy-resistance phenotype [26,27,28,29]. Accordingly, high MT1-MMP expression/function is often correlated with a poor cancer survival prognosis [30,31]. In brain cancer, our knowledge of MT1-MMP expression within tumor grades [32], and its functional impact on LRP-1-mediated ligand internalization within the tumor compartment remains limited.

Interestingly, LRP-1 activity/expression at the cell surface is controlled through intracellular trafficking mechanisms [33] and by shedding processes that involve proteolytic cleavage of LRP-1′s extracellular domain. These are executed by sheddases, among which are the α-secretases ADAM10 and ADAM17, and several membrane-bound MMPs, including MT1-MMP [34,35,36,37,38]. Since LRP-1 is a substrate of MT1-MMP, we specifically questioned here how the MT1-MMP-to-LRP-1 cell surface expression ratio might regulate in vitro LRP-1-mediated An2 internalization within a human glioblastoma cell model.

## 2. Results

### 2.1. LRP-1 and MT1-MMP Gene Expression Profiling in Grade 1–4 Brain Tumor Tissues

**Figure 1:**
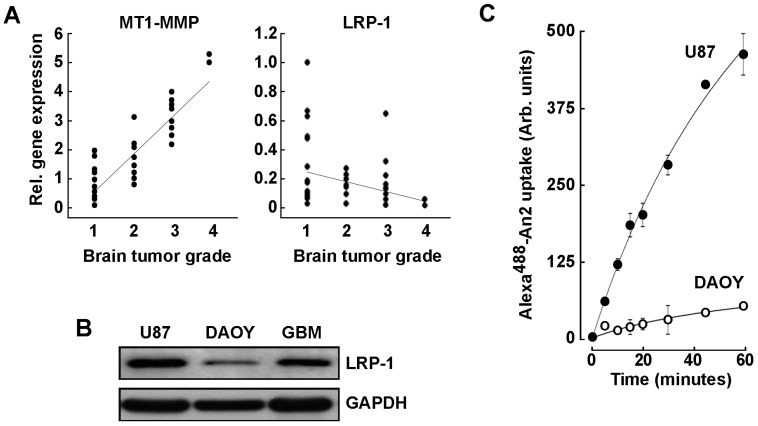
MT1-MMP and LRP-1 transcript levels in grade 1–4 brain tumor tissues. (**A**) TissueScan™ brain cancer and normal tissue cDNA arrays from 43 clinical samples covering four stages of brain cancer were used to assess MT1-MMP and LRP-1 gene expression levels. (**B**) Lysates from adult U87 glioblastoma cells, pediatric DAOY medulloblastoma cells, and from an adult brain tumor tissue were immunodetected for LRP-1 expression. (**C**) Uptake of Alexa^488^-An2 was performed in U87 glioblastoma cells (closed circles) and in DAOY medulloblastoma cells (open circles) as described in the Methods section. Uptake measurements are triplicates from a representative experiment performed out of two.

We first assessed how LRP-1 and MT1-MMP expressions correlate with the malignancy and the invasiveness of the brain tumors as classified by the WHO grading system. Within this system, grade 1 brain tumors are classified as the most benign and slow growing, while grade 4 are the most aggressive tumors [39]. A brain cancer tissue scan cDNA array was used to assess their respective transcript levels. LRP-1 transcript levels appeared to inversely correlate with increasing brain tumor gradation (Figure 1A, right panel). Conversely, MT1-MMP expression increased with the tumor grade in accordance with previous observations which reported high MT1-MMP expression as correlating with poor survival in human GBM patients (Figure 1A, left panel) [40]. In parallel, LRP-1 protein expression was compared between adult U87 glioblastoma cells and pediatric DAOY medulloblastoma cells and was found expressed as much as in a sample of brain tumor tissue homogenate, whereas DAOY cells expressed very low levels of LRP-1 (Figure 1B). Such differential LRP-1 expression was also found to correlate with a higher cell internalization of an LRP-1 peptide ligand An2 (Figure 1C). This suggests that the extent of LRP-1 expression appears to correlate with ligand internalization functions. Given the high LRP-1 transcript levels observed in low-grade brain tumor tissues, our data further indicate that a potential pool of LRP-1 may be available in those tumors, which would still contribute to LRP-1-mediated An2 uptake. Understanding the mechanisms regulating LRP-1-mediated An2 internalization and its relation to MT1-MMP in brain tumors is relevant in optimizing drug delivery strategies for the treatment of GBM.

### 2.2. An2 Internalization in U87 Glioblastoma Cells Is Increased upon MT1-MMP Gene Silencing

**Figure 2:**
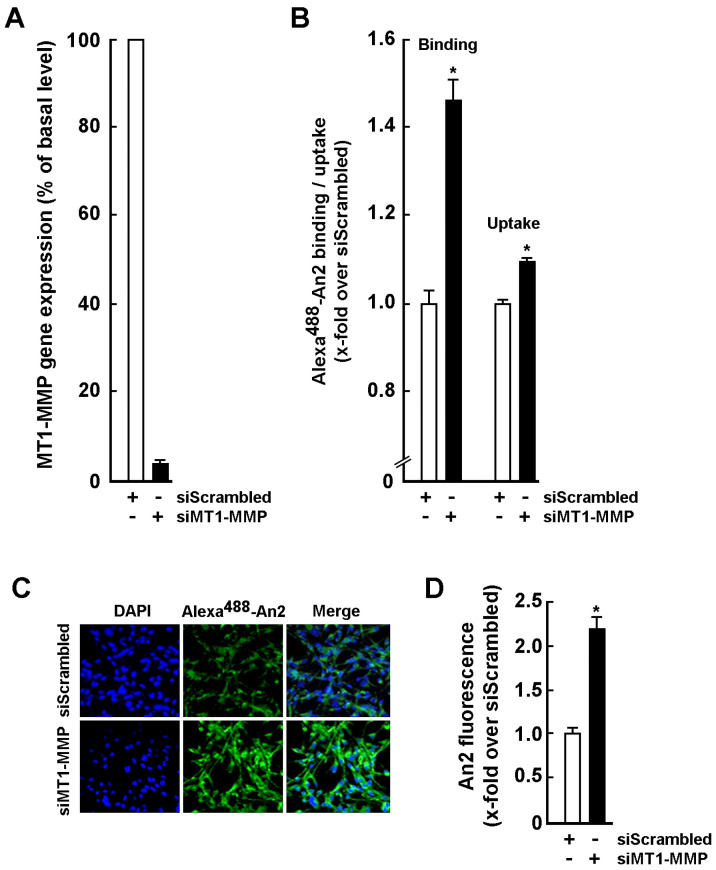
Silencing of MT1-MMP increases An2 cell surface binding and intracellular internalization. (**A**) U87 glioblastoma cells were transiently transfected with 20 nM siRNA (either siScrambled or siMT1-MMP) for 24 h. Total RNA isolation and RT-qPCR were performed as described in the Methods section to assess MT1-MMP gene expression. (**B**) Cells were transiently transfected as in (**A**) and used in cell surface binding or intracellular uptake assays of Alexa^488^-An2 as described in the Methods section. (**C**) Cells were incubated with 100 nM Alexa^488^-An2 for 18 h, fixed and counterstained with DAPI (in blue) to visualize the nuclei. Photomicrographs were taken using confocal microscopy and fluorescence quantified with ImageJ software. 20X magnification. (**D**) Quantification represents the ratio of the mean Alexa^488^-An2 green fluorescence per cell in either siScrambled or siMT1-MMP-transfected cells. Binding/uptake measurements are triplicates from a representative experiment performed out of three independent transfections. Probability values of less than 0.05 were considered significant and an asterisk (*) identifies such significance in the figures.

In order to assess the impact of MT1-MMP cell surface expression on An2 internalization, transient gene silencing was performed with either a siRNA directed against MT1-MMP (siMT1-MMP) or a scrambled siRNA non-specific sequence (siScrambled) in human U87 glioblastoma cells. Cells were then incubated with Alexa^488^-An2 and binding/uptake assays performed as described in the Methods section. Effective silencing of MT1-MMP expression was confirmed by RT-qPCR (Figure 2A) and was correlated with significant increases in both An2 binding and uptake processes (Figure 2B). The increased An2 internalization in siMT1-MMP-transfected cells was further confirmed by confocal microscopy (Figure 2C) as fluorescence quantification shows that suppressing MT1-MMP significantly improved the basal uptake of Alexa^488^-An2 (Figure 2D). These suggest that MT1-MMP exerts a suppressive role in An2 internalization processes and that its inhibition could relieve that suppressive function and possibly lead to improved An2–drug conjugate accumulation within brain tumors.

### 2.3. Increase in Cell Surface MT1-MMP Catalytic Activity Downregulates An2 Internalization

**Figure 3:**
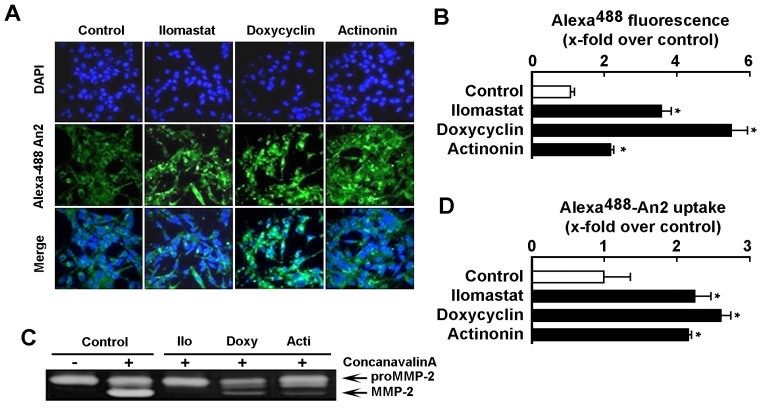
Inhibition of MMP catalytic activity leads to increased An2 intracellular internalization. (**A**) U87 glioblastoma cells were cultured on cover slips, treated with broad-range MMP inhibitors Ilomastat (10 µM), Doxycycline (25 µM) or Actinonin (25 µM) for 24 h in serum-depleted media, incubated with 100 nM of Alexa^488^-An2 for 18 h, fixed and counterstained with DAPI (in blue) to visualize the nuclei. Photomicrographs were taken using confocal microscopy (20X magnification) and (**B**) fluorescence (in green) quantified with ImageJ software. Quantification represents the ratio of the mean green fluorescence per cell. Fluorescence measurements are from four fields taken from a representative experiment performed out of two. (**C**) U87 glioblastoma cells were treated as in (**A**) in combination with 30 µg/mL of Concanavalin-A. Samples from the conditioned media were harvested to perform gelatin zymography in order to assess the extent of MT1-MMP-mediated proMMP-2 activation. (**D**) Intracellular uptake assay of An2 was performed as described in the Section 4 with 100 nM Alexa^488^-An2 for 18 h in the presence or not of the above MMP inhibitors. Uptake measurements are triplicates from a representative experiment performed out of two. Probability values of less than 0.05 were considered significant and an asterisk (*) identifies such significance in the figures.

**Figure 4:**
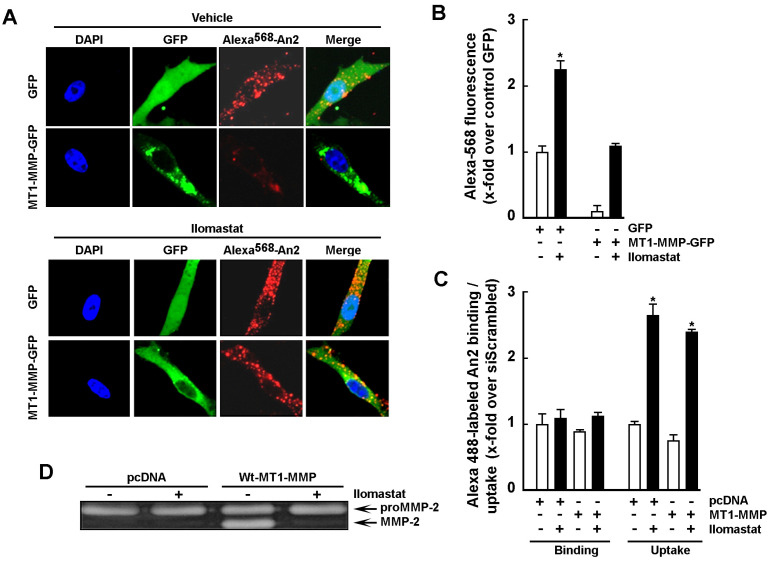
MT1-MMP overexpression decreases An2 internalization. (**A**) U87 glioblastoma cells were transiently transfected with 1 µg of cDNA plasmids encoding either WT-MT1-MMP-GFP or GFP alone, treated with or without 10 µM Ilomastat for 24 h, incubated with 100 nM of Alexa^568^-An2 (in red) for 18 h, fixed and counterstained with DAPI (in blue) to visualize the nuclei. Photomicrographs were taken using confocal microscopy. 40X magnification. (**B**) Fluorescence was quantified with ImageJ software and represents the ratio of the mean red fluorescence per green, fluorescent cell. Fluorescence measurements are from four fields taken from a representative experiment per-formed out of two. (**C**) U87 cells were transiently transfected as in A) with cDNA plasmids encoding either full-length Wt-MT1-MMP or an empty vector (pc DNA3.1). Cells were then treated with or without 10 µM Ilomastat for 24 h and used to perform a binding/uptake of An2 as described in the Methods section. Binding/uptake measurements are triplicates from a representative experiment performed out of two. (**D**) Conditioned media were isolated from serum-starved U87 glioblastoma cells transiently transfected as in (**B**) in order to assess MT1-MMP-mediated activation of latent proMMP-2 into active MMP-2 in the presence or not of Ilomastat. Probability values of less than 0.05 were considered significant and an asterisk (*) identifies such significance in the figures.

Given that decreased MT1-MMP expression led to increased An2 internalization, we next assessed whether increasing MT1-MMP expression or catalytic activity would modulate An2 binding/uptake processes. Pharmacological MMP inhibition using Ilomastat (GM6001), Doxycycline, or Actinonin as well as transient transfection of cells with a cDNA encoding recombinant Wt-MT1-MMP were performed [41]. Using fluorescent microscopy (Figure 3A), we found that all three MMP inhibitors significantly increased Alexa^488^-An2 internalization (Figure 3B). Efficient functional inhibition of MT1-MMP catalytic functions was validated by zymography as MT1-MMP-mediated proMMP-2 activation in Concanavalin-A-treated cells was abrogated (Figure 3C). Uptake of Alexa^488^-An2 was also assessed by flow cytometry in the presence or absence of MMP inhibitors and was increased by all three inhibitors (Figure 3D). Next, recombinant Wt-MT1-MMP-GFP or GFP alone were overexpressed in U87 glioblastoma cells, and subsequently incubated with Alexa^568^-An2 in the presence or absence of MMP inhibitors. Visualization of Wt-MT1-MMP-GFP-positive cells was performed by fluorescent microscopy (Figure 4A) and it was observed that these cells demonstrated less internalization of Alexa^568^-An2 (Figure 4B). Interestingly, Ilomastat eliminated the effect of recombinant MT1-MMP, confirming that the catalytic activity of MT1-MMP was required to alter An2 internalization (Figure 4B). Interestingly, flow cytometry analysis indicated that the binding and uptake of Alexa^568^-An2 were unaffected in cells overexpressing MT1-MMP whether treated or not with Ilomastat (Figure 4C). The differences observed from the results obtained by confocal microscopy versus those obtained by flow cytometry with regard to cells overexpressing Wt-MT1-MMP, can in part be explained by differences in how the analysis are performed. To wit, confocal microscopy allows one to assess the fluorescence associated to a single transfected cell, whereas flow cytometry rather reflects the global fluorescence signal as monitored classically upon 10,000 cell events. Since at best 10–15% of the cells are transfected, the slight decrease, although none statistically significant, in the uptake of An2 in Wt-MT1-MMP condition (Figure 4C) should therefore be considered as confirming the results observed by microscopy (Figure 4B). To validate the functional status of recombinant cell surface MT1-MMP, its extracellular catalytic activity was measured using gelatin zymography to monitor its ability to trigger activation of latent proMMP-2 into active MMP-2 in the cell’s conditioned media. As demonstrated, cells overexpressing recombinant Wt-MT1-MMP led to efficient activation of latent proMMP-2 and this was abrogated by Ilomastat (Figure 4D).

### 2.4. MT1-MMP Catalytic Activity Is Required for Cell Surface Shedding of LRP-1

**Figure 5:**
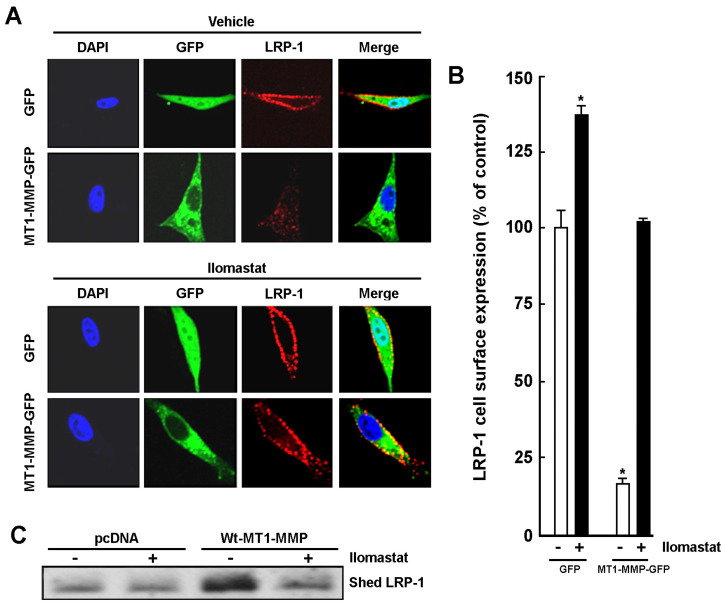
MT1-MMP-mediated shedding of LRP-1 requires MMP catalytic function. (**A**) U87 glioblastoma cells were transiently transfected with 1 µg of cDNA plasmids encoding a GFP-tagged full-length Wt-MT1-MMP or GFP alone. Cells were treated with or without 10 µM Ilomastat for 24 h and fixed. Cell surface LRP-1 labeling was performed in non-permeabilized cells using a LRP-1 heavy chain (515 kDa) primary antibody and a Rhodamine Red-X secondary antibody (in order to display LRP-1 cell surface expression in red). Cells were then counterstained with DAPI (in blue) to visualize the nuclei. Photomicrographs were taken using confocal microscopy. 40X magnification. (**B**) Fluorescence was quantified with the ImageJ software in order to quantify LRP-1 cell surface expression. Cell surface fluorescence measurements are triplicates from a representative experiment performed out of two independent transfections. (**C**) U87 glioblastoma cells were treated as in (**A**). Conditioned media were harvested, and the shed LRP-1 fragment immunodetected. Probability values of less than 0.05 were considered significant and an asterisk (*) identifies such significance in the figures.

In order to confirm the link between MT1-MMP’s catalytic activity and its ability to alter the LRP-1-mediated An2 internalization process, we sought to evaluate MT1-MMP’s ability to shed LRP-1 from the cell surface [42]. U87 glioblastoma cells were transiently transfected with a Mock vector (GFP) or a cDNA encoding the full-length Wt-MT1-MMP-GFP recombinant protein, and then cells were treated with vehicle or Ilomastat. Transfection efficacy was confirmed by visualizing GFP- or Wt-MT1-MMP-GFP-fluorescent cells (Figure 5A, green). LRP-1 cell surface immunolabelling was performed (Figure 5A, red) and, when quantified in green, fluorescent cells, found to be decreased in cells overexpressing Wt-MT1-MMP-GFP (Figure 5B, white bars). Ilomastat significantly prevented that decrease and was also found to increase LRP-1 cell surface expression by 37% in control GFP-transfected cells (Figure 5B, black bars). This observation correlates with data on An2 internalization (Figure 3D) and strengthens the involvement of the MMP-mediated catalytic processes. LRP-1 cell surface shedding into the culture media was also measured to validate the hypothesis that LRP-1 is shed from the cell surface through MT1-MMP-mediated proteolytic activity. Conditioned media were harvested and concentrated. Increased expression of MT1-MMP-mediated LRP-1 immunoreactive cleavage product of ~250 kDa was observed in cells overexpressing recombinant MT1-MMP, whereas this shedding was decreased in Ilomastat-treated cells (Figure 5C). These results in part explain how MT1-MMP cell surface expression/function status may reduce the efficacy of LRP-1-mediated An2 internalization in brain cancer cells.

## 3. Discussion

GBM are characterized by many different genotypic modifications; hence, they are highly heterogeneous and very difficult to treat. The current standard-of-care therapy for GBM includes maximal safe resection followed by concurrent temozolomide and radiation therapy up to 60 Gy. This is expected to produce a maximum median survival of only 14.6 months, as compared to 12.1 months for patients treated with radiation therapy alone [43,44]. Not surprisingly, the pleiotropic biomarker expression profiles further means that GBM patients may likely respond differently to therapies [45]. Here, we provide evidence for differential genotypic MT1-MMP and LRP-1 biomarker expression between tissues from grade 1 to 4 brain tumors. Given the expression profiles recognized for these two biomarkers, our observations imply that, first, a pool of LRP-1 may be inducible or mobilized to contribute to efficient cell surface LRP-1-mediated An2 brain cancer cell internalization and, second, that a given MT1-MMP expression status may possibly dictate either intracellular LRP-1 trafficking to the cell surface or efficient cell surface LRP-1 functions. Given that LRP-1-mediated transport of An2–drug conjugates currently represents one of the most promising brain-penetrating strategies, as demonstrated in clinical studies for the treatment of recurrent malignant gliomas [46], we now highlight the potential implications of MT1-MMP as an LRP-1 functional modulator, and of cell surface MMP status on LRP-dependent An2 recognition in a glioblastoma cell model.

LRP-1 has been reported to be highly expressed in most gliomas, and its silencing found to significantly decrease An2 uptake in vitro in a U87 glioma cell model [21]. However, the expression balance of MT1-MMP from grade I to IV may possibly have important impact on LRP-1-mediated transport and compartmentalization of An2–drug bioconjugates inside the cells. In the current study, we found that MT1-MMP actually contributed to LRP-1 shedding and to reduced cell-surface availability to perform An2 internalization. Considering the binding nature of An2 against LRP-1 ectodomain, it is plausible that the soluble form of LRP-1 would function as the decoy receptor for An2 in the conditioned media and reduce the efficacy of An2 internalization. Further, whether MT1-MMP could cleave any additional cell surface receptors involved in An2 internalization cannot be precluded. In support of this contention, MMP inhibitors such as Ilomastat, Doxycycline and Actinonin all favored the uptake of An2. Inhibiting MT1-MMP cell surface functions may therefore increase the LRP-1-mediated An2 internalization mechanism and, ultimately, that of An2–drug conjugates. Interestingly, pharmacological inhibition or gene silencing of MT1-MMP has been shown to potentiate antiglioma therapies using either combined temozolomide/radiation therapy [47] or glioma virotherapy [48]. It therefore becomes tempting to suggest that the combined use of MMP inhibitors with An2–drug conjugates for high-grade glioma would be beneficial. Moreover, low MT1-MMP-expressing tumor cells, such as low-grade gliomas, might also ultimately be considered as good disease models for efficient An2–drug conjugates treatment.

MT1-MMP involvement has been inferred in the regulation of numerous signaling axes involved in cell invasion, survival and adaptive metabolic control. These include a MT1-MMP/HIF1α [49], MT1-MMP/NFκB [50], MT1-MMP/G6PT [51], MT1-MMP/Akt [52], MT1-MMP/MEK [53], and MT1-MMP/LOX-1 [54]. MT1-MMP and LRP-1 have also been suggested to serve as a new effector-target-molecular axis in the control of PDGF-BB-PDGFRβ-dependent vascular smooth muscle cell phenotype and function [42]. Most of the above MT1-MMP-dependent signaling axes highlight a crucial function of its cytoplasmic domain in intracellular transducing events, and in its dynamin-dependent endocytosis-regulated trafficking in clathrin-coated pits [55]. Given that both MT1-MMP and LRP-1 are dynamically regulated through endocytic processes, and that differential gene expression profiles have been observed among the brain tumor tissues herein assessed (Figure 1), it remains to be established whether and how modulating intracellular trafficking of MT1-MMP and/or LRP-1 to the cell surface of low-grade to high-grade gliomas may affect the LRP-1-mediated An2 internalization process. Interestingly, RAB38, a new member of the RAB small G protein family that regulates intracellular vesicle trafficking [56], RAB27a [57], as well as RAB34 [58] have all been reported to confer a poor prognosis, associated with malignant progression and subtype preference in gliomas.

To date, many studies have documented MT1-MMP’s ability to activate soluble MMPs [59], to transduce intracellular signaling pathways via its cytoplasmic domain [28,50,60,61,62], or to cleave candidate cell-surface receptors that could serve as an anchor for future therapies [21]. Among such cell surface candidates, CD44 is a glycoprotein overexpressed in numerous cancers and which binds to its ligand hyaluronic acid [63]. Although CD44 is reported to be shed from the cell surface by MT1-MMP [62,64], it still was exploited for the creation of HA–drug bioconjugate carriers for selective delivery of various drugs such as paclitaxel, doxorubicin, camptothecin or cisplatin to cancer cells [65,66]. Such observations reinforce the important concept that the cell surface protein expression balance must be crucially taken into account for all receptor-mediated chemotherapy.

With regard to the BBB, after the removal of the tumor as part of standard care, it still may remain a problem for chemo treatment or surgery since the BBB in the periphery of the tumor remains intact and so that the remaining glioma cells be protected in these areas. It is known that brain microvasculature plays a crucial role in sustaining glioblastomas and, as a result, is also a therapeutic target. The BBTB encompasses existing and newly formed blood vessels that contribute to the delivery of nutrients and oxygen to the tumor and facilitate glioma cell migration to other parts of the brain. The high metabolic demands of high-grade glioma create hypoxic areas that trigger increased expression of VEGF and angiogenesis, leading to the formation of abnormal vessels and a dysfunctional BBTB. Even though the BBTB is considered ‘leaky’ in the core part of glioblastomas, in large parts of glioblastomas and, even more so, in lower grade diffuse gliomas the BBTB will more closely resemble the intact BBB and prevent efficient passage of cancer therapeutics, including small molecules and antibodies. Thus, many drugs can still be blocked from reaching the many infiltrative glioblastoma cells. Given LRP-1 is highly expressed within such tumor microenvironment provides the rational for An2 conjugates to overcome the BBTB.

**Figure 6:**
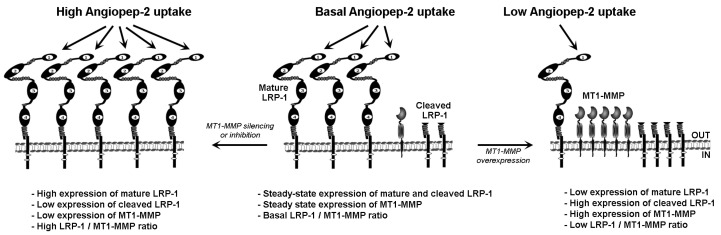
LRP-1/MT1-MMP expression ratio dictates An2 internalization capacity. Scheme summarizing the functional impact on the An2 intracellular internalization process of a high LRP-1/MT1-MMP ratio obtained through MT1-MMP silencing or pharmacological inhibition (left, high An2 uptake), a basal LRP-1/MT1-MMP ratio (middle, basal An2 uptake), and a low LRP-1/MT1-MMP ratio (right, low An2 uptake).

A scheme of the molecular signature characterizing models of low MT1-MMP/LRP-1 expression ratio versus high MT1-MMP/LRP-1 expression ratio is depicted (Figure 6). We hypothesize that low MT1-MMP-to-LRP-1 ratios or MT1-MMP inhibition would ultimately favor LRP-1-mediated internalization of An2–drug conjugates in gliomas. MMP inhibitors in cancer therapy, potentially through combination treatments with cytotoxics, are at a turning point past failures into future successes [67]. A first step may be to perform “window of opportunity” trials in early-stage cancers, identifying and validating biomarkers of enzymatic inhibition and metastasis as proxy for clinical success.

## 4. Materials and Methods

### 4.1. Materials

Sodium dodecylsulfate (SDS) and bovine serum albumin (BSA) were purchased from Sigma-Aldrich Canada (Oakville, ON, Canada). Cell culture media were obtained from Life Technologies (Burlington, ON, Canada). Electrophoresis reagents were purchased from Bio-Rad (Mississauga, ON, Canada). The HyGLO chemiluminescent HRP antibody detection reagent was from Denville Scientific Inc. (Metuchen, NJ, USA). Micro bicinchoninic acid protein assay reagents were from Pierce (Rockford, IL, USA). The MMP inhibitor Ilomastat was from Merck Millipore (Etobicoke, ON, Canada), while Doxycycline and Actinonin were from Sigma-Aldrich Canada. Angiopep-2 (An2), Alexa^488^-An2, and Alexa^568^-An2 were a gift from Angiochem Inc (Montreal, QC, Canada). The monoclonal anti-MT1-MMP catalytic domain antibody 3G4.2 was from EMD Millipore (Billerica, MA, USA). The anti-LRP Heavy Chain mouse antibody (8G1) was from Calbiochem (San Diego, CA, USA). The PE mouse anti-human CD91 (LRP-1) antibody and PE mouse IgG1 κ isotype control were purchased from BD Biosciences (Mississauga, ON, Canada). Horseradish peroxidase-conjugated donkey anti-rabbit and anti-mouse IgG secondary antibodies were from Jackson ImmunoResearch Laboratories (West Grove, PA, USA). Ultracel 10 K centrifugal filters were from Merck Millipore (Etobicoke, ON, Canada). All other reagents were from Sigma-Aldrich Canada.

### 4.2. Cell Culture

The human U87 glioblastoma (HTB-14) and DAOY medulloblastoma cell lines were from American Type Culture Collection (Manassas, VA, USA). They were both maintained in Eagle’s Minimum Essential Medium (Wisent, 320-006CL) containing 10% (*v*/*v*) calf serum (HyClone Laboratories, SH30541.03), 2 mM glutamine, 1 mM sodium pyruvate (Sigma-Aldrich, Oakville, ON, Canada, P2256), 100 units/mL penicillin and 100 mg/mL streptomycin (Wisent, 250-202-EL). Cells were incubated at 37 °C with 95% air and 5% CO_2_.

### 4.3. TissueScan cDNA Arrays of Grades I-IV Brain Tumor Tissues

TissueScan^TM^ cancer and normal tissue cDNA arrays were purchased from OriGene (Rockville, MD, USA), covering 43 clinical samples of the four stages of brain cancer as well as normal tissues, and were used to assess MT1-MMP and LRP-1 gene expression levels according to the manufacturer’s recommendations. Tissue cDNAs in each array were synthesized from high quality total RNAs of pathologist-verified tissues, normalized and validated with β-actin in two sequential qPCR analyses, and accompanied by clinical information for eighteen WHO grade 1, eleven WHO grade 2, ten WHO grade 3, and two WHO grade 4 brain tumors (https://www.origene.com/products/tissues/tissuescan; accessed on 1 August 2022). Detailed clinical information can be found in the Supplementary file appended.

### 4.4. Angiopep-2 Binding and Uptake Assays Using flow Cytometry

Cells were incubated with 100 nM Alexa^488^-An2/Ringer-HEPES or Ringer-HEPES alone for 1 h at 4 °C (Binding) or at 37 °C (Uptake) in the dark, and washed three times with PBS/BSA(5%)/EDTA(2nM). Fluorescence levels were then assessed by flow cytometry in the FL1-A channel using a BD Biosciences C6 Accuri flow cytometer (Mississauga, ON, Canada).

### 4.5. Binding and Uptake Assays of Angiopep-2 Assessed by Confocal Microscopy

Cells were incubated with 50 nM Alexa^488^-An2 or Alexa^568^-An2/EMEM medium without phenol red for 18 h at 37 °C and 5% CO_2_. Cells were fixed in 4% formaldehyde (Fisher Scientific, Ottawa, ON, Canada) for 20 min. A solution of 2 μg/mL DAPI (Invitrogen, Waltham, MA, USA, #D1306) diluted in PBS was used to stain the nuclei for 5 min and mounted onto slides using Prolong Gold antifade reagent (Invitrogen, Waltham, MA, USA, P36934). Fluorescence was then monitored by confocal microscopy with a Nikon Eclipse Ti confocal microscope and quantified with the ImageJ analysis software.

### 4.6. Immunofluorescent Microscopy

U87 glioblastoma cells were cultured on cover slips, transiently transfected with 1 µg of cDNA plasmid encoding either a full-length GFP-tagged Wt-MT1-MMP recombinant protein (Wt-MT1-MMP-GFP) or a GFP recombinant protein (GFP) and treated (or not) with 25 µM Ilomastat. Cells were serum-starved for 24 h. Media were collected and cells were fixed in 4% formaldehyde for 20 min then blocked for 1 h in 1% BSA/PBS/NaN_3_. Immunostaining was performed under non-permeabilizing conditions for 1 h with the anti-LRP Heavy Chain antibody (2 µg/mL) in 1% BSA/PBS/NaN_3_, followed by 1:200 Rhodamine Red-X donkey anti-mouse IgG (Jackson ImmunoResearch Inc, West Grove, PA, USA). A solution of 10 µg/mL DAPI diluted in PBS was used to stain the nuclei. Fluorescence was then examined by confocal microscopy on a Nikon Eclipse Ti confocal microscope and quantified using ImageJ analysis software.

### 4.7. Measurement of LRP-1 Cell-Surface Expression

The cells were collected upon PBS/Citrate (1 mM) treatment, resuspended in a solution of binding buffer and incubated with either the PE mouse anti-human CD91 (LRP-1) antibody or PE mouse IgG1 κ isotype control for 1 h at room temperature in the dark. Cells were washed three times with PBS then processed with a direct flow cytometry analysis in the FL2-A channel with a C6 Accuri flow cytometer to evaluate LRP-1 cell surface expression. The results obtained were quantified as a ratio of the GeoMeans of LRP-1-PE over control PE antibodies.

### 4.8. Gelatin Zymography

Assessment of the extracellular hydrolytic levels of latent proMMP-2 and active MMP-2 was performed by gelatin zymography. Briefly, an aliquot (20 μL) of the culture medium was subjected to SDS-polyacrylamide gel electrophoresis (SDS-PAGE) in a gel containing 0.1 mg/mL gelatin (Sigma-Aldrich, Oakville, ON, Canada). The gels were then incubated in 2.5% Triton X-100 (Bioshop, TRX506.500) and rinsed in nanopure distilled water. Gels were further incubated at 37 °C for 20 h in 20 mM NaCl, 5 mM CaCl_2_, 0.02% Brij-35, 50 mM Tris-HCl buffer, pH 7.6 and then stained with 0.1% Coomassie Brilliant Blue R-250 (Bioshop, CBB250) and destained in 10% acetic acid and 30% methanol in water. Gelatinolytic activity was detected as unstained bands on a blue background.

### 4.9. Total RNA Isolation, cDNA Synthesis and Real-Time Quantitative RT-PCR

Total RNA was extracted from cell monolayers using TriZol reagent (Life Technologies, Carlsbad, CA, USA, 15596-018). For cDNA synthesis, 2 μg of total RNA were reverse transcribed using a high capacity cDNA reverse transcription kit (Applied Biosystems, Waltham, MA, USA, 4368814). cDNA was stored at −80 °C prior to PCR. Gene expression was quantified by real-time quantitative PCR using Sso Fast EvaGreen Supermix (Bio-Rad). DNA amplification was carried out using a CFX connect Real-Time System (Bio-Rad) and product detection was performed by measuring binding of the fluorescent dye EvaGreen to double-stranded DNA. The following QuantiTect primer sets were provided by QIAGEN: LRP-1 (Hs_LRP1_1_SG QT00025536), MT1-MMP (Hs_Mmp14_1_SG QT00001533), GAPDH (Hs_GAPDH_2_SG QT01192646), β-actin (Hs_Actb_2_SG QT01680476) and PPIA (Hs_PPIA_4_SG QT01866137). The relative quantities of target gene mRNA compared against two internal controls chosen from GAPDH, β-actin or PPIA RNA, were measured by following a ΔCT method employing an amplification plot (fluorescence signal vs. cycle number). The difference (ΔCT) between the mean values in the triplicate samples of target gene and those of GAPDH and β-actin mRNAs were calculated by CFX manager Software version 2.1 (Bio-Rad) and the relative quantified value (RQV) was expressed as 2^-ΔCT^.

### 4.10. Transfection Method and RNA Interference

Cells were transiently transfected with 1 µg of cDNA plasmids encoding either WT-MT1-MMP, WT-MT1-MMP-GFP, GFP or empty vector for 24 h. Validation of MT1-MMP overexpression was performed upon monitoring of proMMP-2 activation by gelatin zymography. For gene silencing experiments, cells were transiently transfected with 20 nM siRNA against MT1-MMP (Hs_MMP14_6 HP validated siRNA; QIAGEN, SI03648841) or scrambled sequences (AllStar Negative Control siRNA; QIAGEN, 1027281) using Lipofectamine 2000 (Invitrogen, 11668). MT1-MMP-specific gene silencing efficacy was assessed by RT-qPCR as described above.

### 4.11. Immunoblotting Procedures

Primary human brain tumors were obtained from Notre-Dame Hospital (Montreal, QC, Canada). Proteins (20 μg) from tissue homogenate, control cells, and treated cells were separated by SDS-PAGE. After electrophoresis, proteins were electrotransferred to polyvinylidenedifluoride membranes which were then blocked for 1 h at room temperature with 5% non-fat dry milk in Tris-buffered saline (150 mM NaCl, 20 mM Tris–HCl, pH 7.5) containing 0.3% Tween-20 (TBST). Membranes were further washed in TBST and incubated with the primary antibodies (1/1000 dilution) in TBST containing 3% BSA and 0.1% sodium azide, followed by a 1 h incubation with horseradish peroxidase-conjugated anti-rabbit or anti-mouse IgG (1/5000 dilution) in TBST containing 5% non-fat dry milk. Immunoreactive material was visualized by ECL (Amersham Biosciences, Baie d’Urfé, QC, Canada).

### 4.12. Statistical Data Analysis

Data are representative of triplicate measurements of two to three independent experiments, and represented as the means +/− SEM. Statistical significance was assessed using Student’s unpaired t-test or one-way ANOVA with a Dunnett post-test. Probability values of less than 0.05 were considered significant and an asterisk identifies such significance in the figures.

## 5. Conclusions

This study strengthens the involvement of LRP-1 in An2-mediated internalization processes taking place in brain cancer cells. Our study further suggests that regulation of LRP-1 cell surface expression and function, through gene silencing or pharmacological inhibition of catalytic MMP activity, might become very useful in enhancing the chemotherapeutic effects of An2–drug conjugates [46,68,69] by promoting their internalization in both low-grade- and high-grade gliomas. How the MMP inter-relation with LRP-1 may alter future clinical initiatives remains to be explored. Recently, An2-decorated nanostructures have been employed to deliver a vast array of therapeutic and diagnostic agents. These include chemical compounds; the nucleic acids DNA, siRNA, and miRNA; photosensitizers; and contrast agents. In addition, there is a growing body of in vivo data to support the further design of multifunctional An2-modified nanomedicines in immunotherapy, and a great need to translate the in vitro and in vivo achievements of BBB-crossing nanotherapeutics to the clinic.

## Data Availability

All data and used materials are available upon request from the corresponding author.

## Supplementary Materials

The supporting information can be downloaded at: https://www.mdpi.com/article/10.3390/ijms232214214/s1.
